# Producing air-stable monolayers of phosphorene and their defect engineering

**DOI:** 10.1038/ncomms10450

**Published:** 2016-01-22

**Authors:** Jiajie Pei, Xin Gai, Jiong Yang, Xibin Wang, Zongfu Yu, Duk-Yong Choi, Barry Luther-Davies, Yuerui Lu

**Affiliations:** 1 Research School of Engineering, College of Engineering and Computer Science, the Australian National University, Canberra, Australian Capital Territory 2601, Australia; 2 School of Mechanical Engineering, Beijing Institute of Technology, Beijing 100081, China; 3 CUDOS, Laser Physics Centre, Research School of Physics and Engineering, the Australian National University, Canberra, Australian Capital Territory 2601, Australia; 4 Department of Electrical and Computer Engineering, University of Wisconsin, Madison, Wisconsin 53706, USA

## Abstract

It has been a long-standing challenge to produce air-stable few- or monolayer samples of phosphorene because thin phosphorene films degrade rapidly in ambient conditions. Here we demonstrate a new highly controllable method for fabricating high quality, air-stable phosphorene films with a designated number of layers ranging from a few down to monolayer. Our approach involves the use of oxygen plasma dry etching to thin down thick-exfoliated phosphorene flakes, layer by layer with atomic precision. Moreover, in a stabilized phosphorene monolayer, we were able to precisely engineer defects for the first time, which led to efficient emission of photons at new frequencies in the near infrared at room temperature. In addition, we demonstrate the use of an electrostatic gate to tune the photon emission from the defects in a monolayer phosphorene. This could lead to new electronic and optoelectronic devices, such as electrically tunable, broadband near infrared lighting devices operating at room temperature.

## Abstract (web summary)

It has been a long-standing challenge to produce air-stable few- or monolayer phosphorene. Here, the authors demonstrate a new highly controllable method for fabricating high quality, air-stable mono- and few-layer phosphorene films, enabling precise defect engineering in monolayer phosphorene.

## Graphical abstract

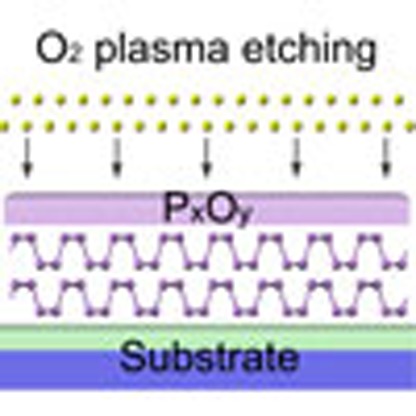

Phosphorene, a recently developed two-dimensional (2D) material, has attracted tremendous interest because of its unique properties, including its anisotropic nature^1^,^2^,^3^,^4^,^5^,^6^,^7^; its layer-dependent direct bandgap energy^8^,^9^; and its quasi-one-dimensional excitonic nature^10^,^11^. These properties contrast markedly with those of other 2D materials, such as graphene^12^ and monolayer transition-metal dichalcogenide semiconductors^13^,^14^,^15^,^16^,^17^. In particular, a monolayer of phosphorene provides a unique 2D platform for investigating the dynamics of excitons in reduced dimensions and fundamental many-body interactions^10^,^18^,^19^, and this could lead to many novel electronic and optoelectronic devices^1^,^2^,^3^,^4^,^5^,^6^,^8^,^9^,^10^,^11^. However, these unique 2D materials are unstable and degrade very rapidly in ambient conditions^9^,^19^,^20^,^21^. Some recent passivation techniques have been successfully used to stabilize phosphorene flakes, such as coating with Al_2_O_3_ by atomic layer deposition (ALD)^22^,^23^,^24^,^25^ and sandwich encapsulation of phosphorene between hexagonal boron nitride layers^26^,^27^,^28^. However, ALD cannot be used for the passivation of a monolayer or even few-layer (< 5 layers) phosphorene samples, because such thin phosphorene layers are too unstable^19^,^20^,^29^ and are rapidly oxidized by the ALD process itself. Furthermore, the boron nitride encapsulation method requires very complicated environmental conditions and has extremely low throughput and yield^26^,^27^,^28^.

Here we demonstrate a new method for controllably fabricating air-stable and high-quality phosphorene samples with a designated number of layers, through layer-by-layer thinning of thick exfoliated phosphorene flakes using oxygen plasma etching, followed by further passivation with an Al_2_O_3_ coating. Moreover, this fabrication technique has allowed us to precisely engineer the defects in a phosphorene monolayer, and this not only triggers new photon emissions, but also modulates the doping level in the material. In addition, the defect-induced photon emission can itself be modulated using an electric field. Our fabrication technique is a straightforward and efficient process that opens up new avenues for the production of air-stable and high-quality few- or monolayer phosphorene samples. It makes the black phosphorus a practical material for optoelectronic device and fundamental research, while the capability of atomic scale defect engineering in phosphorene achievable by using this approach can also lead to new electronic and optoelectronic devices.

## Results

### Producing air-stable phosphorene

**Figure 1:**
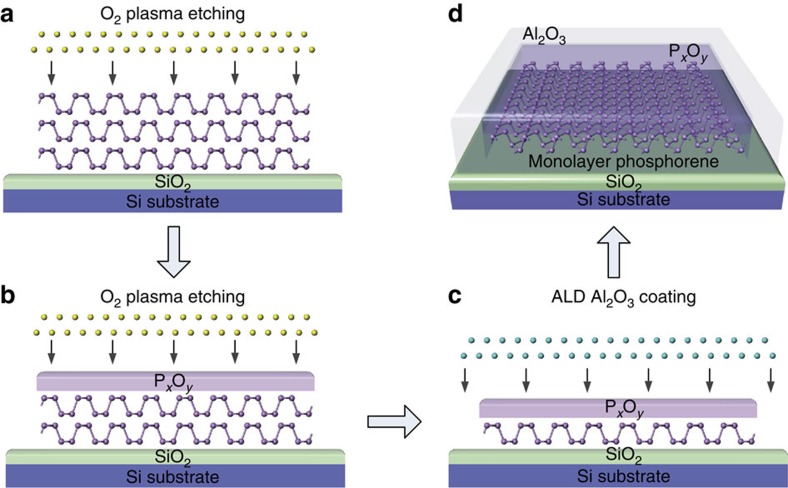
Schematically showing the fabrication of air-stable mono- and few-layer phosphorene samples. (**a**) A thick phosphorene flake is firstly exfoliated onto a SiO_2_/Si substrate and the sample is then treated with O_2_ plasma etching (yellow balls). (**b**) During the O_2_ plasma pre-treatment process, the top layers of the phosphorene flake are oxidized to become P_*x*_O_*y*_, which then serves as a protective layer for the remaining phosphorene sample underneath. With further O_2_ plasma etching, oxygen plasma can penetrate the P_*x*_O_*y*_ layer by diffusion and oxidize the underlying phosphorene, and this thins down the phosphorene layer and also increases the thickness of the P_*x*_O_*y*_. Meanwhile, the O_2_ plasma physically sputters away the P_*x*_O_*y*_ layer from the top because of the collisions by oxygen plasma. After the plasma pre-treatment, a dynamic equilibrium is reached between oxidation of the phosphorene and physical removal of the P_*x*_O_*y*_ layer, such that the P_*x*_O_*y*_ layer approaches a constant thickness and the etching rate also becomes constant. (**c**) Because of the constant etching rate, any designated number of layers of phosphorene down to a monolayer can then be precisely fabricated and the degradation of the remaining layers is inhibited because of the protective nature of the P_*x*_O_*y*_. (**d**) To further improve the lifetime of the phosphorene so-produced, the sample was also coated with an Al_2_O_3_ protective layer by ALD. In this case the P_*x*_O_*y*_ layer prevents the underlying phosphorene from reacting with the precursor gases used in the ALD process, which is especially important for samples less than a few layers thick.

Figure 1 is the schematic showing layer-by-layer thinning of a phosphorene flake by O_2_ plasma etching and the further passivation procedure used to controllably produce air-stable few- or monolayers of phosphorene. A thick phosphorene flake is firstly mechanically exfoliated onto a SiO_2_/Si substrate and the sample is then treated with O_2_ plasma etching (Fig. 1a). During the O_2_ plasma pre-treatment process, the top layers of the phosphorene flake are oxidized to become P_*x*_O_*y*_, which then serves as a protective layer for the remaining phosphorene sample underneath. With further O_2_ plasma etching, oxygen plasma can penetrate the P_*x*_O_*y*_ layer and oxidize the underlying phosphorene, and this thins down the phosphorene layer and also increases the thickness of the P_*x*_O_*y*_. Meanwhile, the O_2_ plasma physically sputters away the P_*x*_O_*y*_ layer from the top because of the collisions by oxygen plasma. After the plasma pre-treatment, a dynamic equilibrium is reached between oxidation of the phosphorene and physical removal of the P_*x*_O_*y*_ layer, such that the P_*x*_O_*y*_ layer approaches a constant thickness and the etching rate also becomes constant (Fig. 1b). Because of the constant etching rate, any designated number of layers of phosphorene down to a monolayer can then be precisely fabricated (Fig. 1c) and the degradation of the remaining layers is inhibited because of the protective nature of the P_*x*_O_*y*_. However, to further improve the durability of the phosphorene so-produced, the sample was also coated with an Al_2_O_3_ protective layer by ALD. In this case the P_*x*_O_*y*_ layer prevents the underlying phosphorene from reacting with the precursor gases used in the ALD process, which is especially important for samples less than a few layers thick (Fig. 1d).

**Figure 2:**
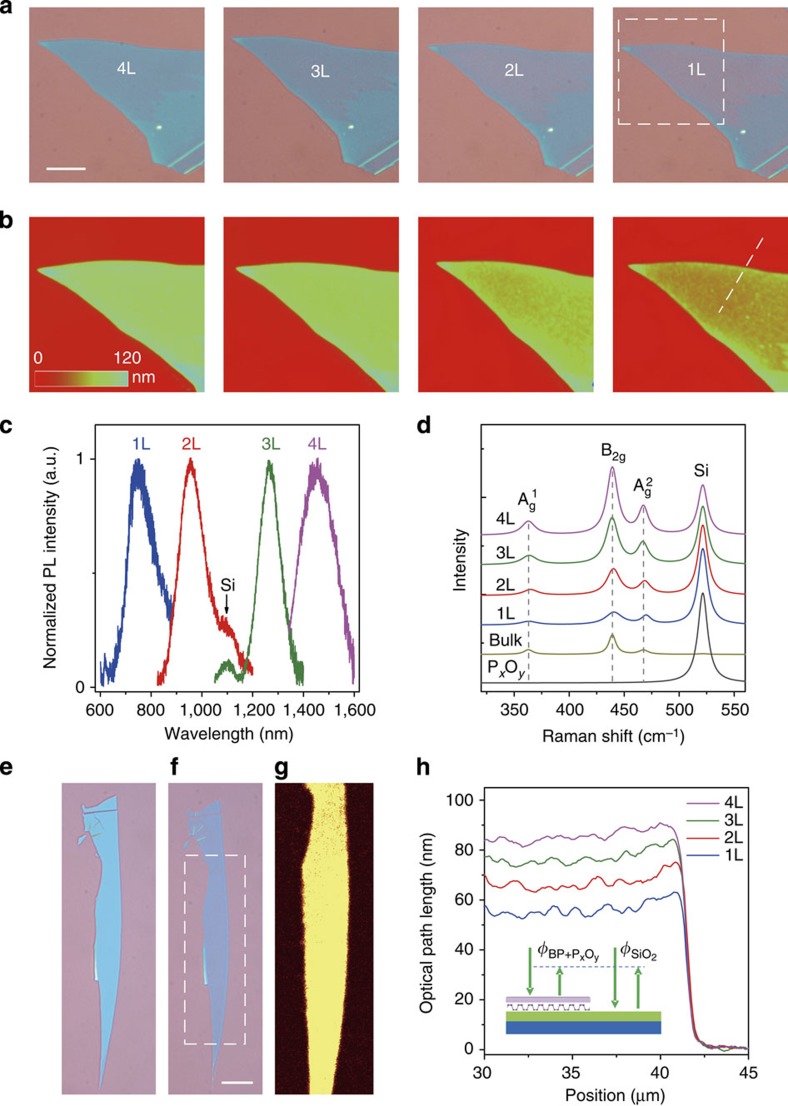
Experimental demonstration of the controllable production of few- and monolayer phosphorene samples by O_2_ plasma thinning. (**a**,**b**) Optical microscope (**a**) and phase-shifting interferometry (PSI) (**b**) images of the phosphorene flake, as it was thinned layer-by-layer down to monolayer (1L). PSI images show the area marked by a dashed square in **a**. Scale bar, 10 μm. (**c**,**d**) Measured photoluminescence (PL) (**c**) and Raman (**d**) spectra from the thin phosphorene samples (quad- to monolayer, 4–1L), fabricated by O_2_ plasma etching. Raman spectra of bulk phosphorene and pure P_*x*_O_*y*_ are also measured and plotted for comparison. The vertical dashed lines are added as eye guidance. The excitation polarization angle was randomly selected for this Raman spectra measurement. (**e**–**g**), Uniformity characterization of the samples produced by O_2_ etching; (**e**,**f**) optical microscopes of a thick phosphorene flake when it was exfoliated (**e**) and thinned down to 2L (**f**) by O_2_ plasma etching. Scale bar, 10 μm. (**g**) Integrated PL mapping image of the area marked by a dashed rectangle in **f**. (**h**) PSI measured OPL values versus position for the plasma-produced 1L, 2L, 3L and 4L phosphorene along the dashed line in **b**. Inset is the schematic plot showing the PSI measured phase shifts of the reflected light from the phosphorene/P_*x*_O_*y*_ stack (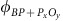) and the SiO_2_ substrate (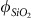).

Figure 2 shows our experimental demonstration for the controllable production of few- and monolayer phosphorene samples by O_2_ plasma thinning. In the experiments, after a pre-treatment of ∼40 s by O_2_ plasma, the phosphorene etching rate reached a constant value of ∼10 s per layer. The capping thickness of the P_*x*_O_*y*_ layer also reached a constant value of ∼11 nm, which was confirmed by experimental measurements (discussed later). The optical microscope images (Fig. 2a) clearly indicates the reduction of the flake contrast, as it was thinned layer-by-layer down to a monolayer. The number of the remaining layers of phosphorene was precisely measured by phase-shifting interferometry (PSI; Fig. 2b) and photoluminescence (PL) spectroscopy (Fig. 2c), which have been demonstrated to be very robust ways of determining the number of layers of exfoliated phosphorene flakes^19^. From Fig. 2c, the measured PL spectra from the phosphorene samples between 1 and 4 layers (1–4L) thick produced by O_2_ plasma etching peaked at 750, 970, 1,290 and 1,440 nm, respectively, corresponding to energies of 1.65, 1.28, 0.96 and 0.86 eV, which matches very well with our previously reported values for exfoliated phosphorene samples^9^,^19^. Also, the measured Raman spectra (Fig. 2d) from 1 to 4L of phosphorene produced by O_2_ plasma etching also match very well with those for mechanically exfoliated samples^29^,^30^. The strongly angle-dependent responses of the A_g_^1^, B_2g_ and A_g_^2^ modes (Supplementary Fig. 1 and Supplementary Note 1) confirms the anisotropic puckered crystalline structure of the monolayer phosphorene samples produced by our etching method^4^,^9^,^20^,^31^. Although there is no defect peak in the Raman spectra of phosphorene, the integrated intensity ratio A_g_^1^/A_g_^2^ reflects the oxidation-induced defect density^29^. Pristine monolayer phosphorene^29^ prepared in a glovebox (no oxidation) has a ratio A_g_^1^/A_g_^2^ of ∼0.4, while partially oxidized samples have a ratio smaller than 0.2. This ratio typically becomes smaller when the sample has more oxidation-induced defects^29^. Our monolayer phosphorene samples produced by O_2_ plasma have integrated intensity ratio A_g_^1^/A_g_^2^ values in the range of 0.37–0.42, which is comparable to the value of the pristine monolayer phosphorene prepared in a glovebox^29^. It further confirms the high quality of our monolayer phosphorene samples produced by O_2_ plasma etching. Also, this A_g_^1^/A_g_^2^ ratio is not that sensitive to the polarization angle^29^, which can also been seen from our measurements (Supplementary Fig. 1b). In addition, the integrated PL mapping image shows that the samples produced by O_2_ plasma etching are extremely uniform (Fig. 2e–g). From these PL and Raman characteristics, we clearly see that the O_2_ plasma etching process can produce large, high quality, single-crystalline phosphorene layers. Compared with the previously reported Ar plasma thinning technique^30^,^32^, our O_2_ plasma thinning process provides a huge advantage by simultaneously producing the P_*x*_O_*y*_ capping layer, which is very critical for the following reasons: (1) the P_*x*_O_*y*_ capping layer serves as a protective coating, on which we can add extra passivation layers such as ALD Al_2_O_3_ or other coatings, leading to the production of air-stable few-layer and monolayer phosphorene films (discussed later); (2) the P_*x*_O_*y*_ capping layer also helps to reduce the etching rate to a very controllable range, which leads to a precise phosphorene/P_*x*_O_*y*_ interface, thus preserving the high quality of the samples; (3) the P_*x*_O_*y*_ capping layer also allows us to precisely engineer the defects in monolayer phosphorene at the atomic scale, triggering new applications in optoelectronic devices (discussed later).

We used the rapid, non-invasive and highly accurate PSI method to quantify the evolution of this important P_*x*_O_*y*_ capping layer during the thinning process (Fig. 2b,h). Specifically, we measured the optical path length (OPL) of the light reflected from the phosphorene/P_*x*_O_*y*_ stack. The OPL is determined as 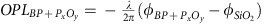, where *λ*=535 nm is the wavelength of the light source, and 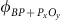 and 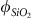 are the phase shifts of the light reflected from the phosphorene/P_*x*_O_*y*_ stack and the SiO_2_/Si substrate (Fig. 2h inset), respectively. The measured average and s.d. error of the OPL values from 0, 1, 2, 3 and 4L phosphorene/P_*x*_O_*y*_ stack samples were (42±2.0), (54±1.5), (64±1.3), (74±1.2) and (84±1.2) nm, respectively (Supplementary Fig. 2), and are independent of the thickness of the initial phosphorene flake before etching. This confirms our previous model that the P_*x*_O_*y*_ layer has constant thickness once the etching reaches the dynamic equilibrium state after the pre-treatment process. Through the theoretical fitting, the thickness of this P_*x*_O_*y*_ capping layer was extracted to be ∼11 nm, which matches well with our atomic force microscopy measurement (Supplementary Figs 2 and 3 and Supplementary Note 2). The thickness of P_*x*_O_*y*_ does vary slightly as a function of the plasma parameters, such as the oxygen flow rate and generator power (Supplementary Fig. 4 and Supplementary Note 3). This is because the number of the neutral species, that dominate the oxidization process, and the number of oxygen ions, that are critical for sputtering, vary at different rates as a function of generator power and flow rate. On the basis of our X-ray photoelectron spectroscopy analysis (Supplementary Fig. 5), the composition of the oxidized phosphorus capping layer is dominated by saturated–oxidized P_2_O_5_ rather than partially oxidized phosphorus^33^,^34^. The saturated–oxidized P_2_O_5_ is the most stable among the phosphorus oxides (P_*x*_O_*y*_) indicating it should provide the best protection as a capping layer. We found that the O_2_ plasma results in ‘step-wise' etching (Supplementary Fig. 6 and Supplementary Note 4), which is related to the diffusion kinetics of oxygen in this P_*x*_O_*y*_ capping layer^35^. This ‘digitized' fabrication process implies great stability and tolerance of the thinning process. In addition, the P_*x*_O_*y*_ capping layer would not affect applications as field effect transistor devices, since the edge contact technique has been demonstrated to be an efficient way for encapsulated 2D materials^36^.

**Figure 3:**
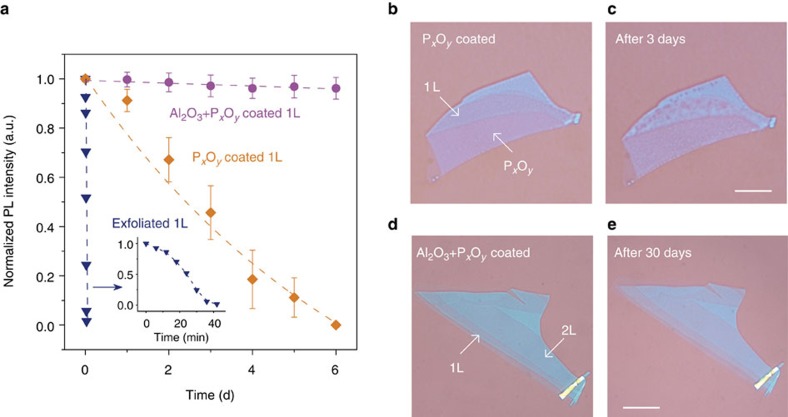
Stability comparison of the phosphorene samples by different fabrication methods. (**a**) Time dependence of the PL intensity of the monolayer phosphorene samples by different methods: exfoliated 1L phosphorene (blue), 1L phosphorene with P_*x*_O_*y*_ (∼11 nm) capping layer produced by O_2_ plasma etching (orange) and 1L phosphorene with dual passivation layers of P_*x*_O_*y*_ and 5 nm of ALD Al_2_O_3_ (pink). All PL was measured in ambient condition under the same laser excitation. The maximum PL intensity is normalized to 1. The error bars represent the measurement variation from at least three different locations on the sample flake. Inset is the zoom in plot for the exfoliated 1L phosphorene sample. (**b**,**c**) Optical microscope images of a 1L phosphorene sample with P_*x*_O_*y*_ capping layer only, when it was produced by O_2_ plasma etching (**b**) and after 3 days (**c**). Scale bar, 10 μm. (**d**,**e**) Optical microscope images a phosphorene sample with two capping layers of P_*x*_O_*y*_ and 5 nm of ALD Al_2_O_3_, when it was just produced by O_2_ plasma etching and ALD further passivation (**d**) and after 30 days (**e**). Scale bar, 10 μm.

The rapid degradation of phosphorene samples in ambient conditions, caused by the photoactivated oxidation by aqueous oxygen^29^, has been a significant hurdle for the investigation of material properties and the exploration of device applications. Our oxygen plasma thinning technique provides an elegant way to solve this problem. The simultaneous production of the P_*x*_O_*y*_ capping layer by O_2_ plasma treatment serves as a protective layer, on which we can add extra passivation layers, such as ALD Al_2_O_3_ and/or other coatings, and as we show next, this leads to the production of air-stable few- and monolayers of phosphorene. In our experiments, the few- and monolayers of phosphorene capped with P_*x*_O_*y*_ exhibited much longer lifetime than exfoliated samples in ambient conditions. We measured the time evolution of the PL intensity from phosphorene monolayer samples fabricated by different methods, as shown in Fig. 3a. In this PL tracking experiment, all the samples were exposed to air at room temperature, with the same laser excitation. The exfoliated monolayers of phosphorene only survived for ≈30 min (Fig. 3a inset), which is consistent with our previous observations^19^. In contrast, the monolayers fabricated by O_2_ plasma etching had much longer lifetime than the exfoliated samples, because the P_*x*_O_*y*_ capping layer protects them from oxygen and moisture (Fig. 3a). However, the P_*x*_O_*y*_ layer itself is not sufficiently robust for complete passivation, since the O_2_ and moisture in the air can erode it slowly, as shown in Fig. 3b,c. Three days after it was produced, the sample with the P_*x*_O_*y*_ capping layer started to degrade and a number of holes were found on the surface (Fig. 3c), and this was accompanied by a drop in the PL intensity (Fig. 3a). After 3 days, the degradation process accelerated and the luminescence completely disappeared after another 3 days. Therefore, an even more robust protective coating is necessary. ALD Al_2_O_3_ is one of the most popular passivation materials that is widely used for photonic devices^37^ and thick phosphorene flakes^22^,^23^,^24^. The Al_2_O_3_ ALD process cannot be directly applied to thin freshly exfoliated phosphorene layers, especially for mono- and bi-layers, because the samples are oxidized by the ALD process itself. However, we found that mono- and bi-layer phosphorene samples fabricated by O_2_ plasma etching can be coated with ALD Al_2_O_3_ without any damage to the underlying phosphorene because of presence of the P_*x*_O_*y*_ capping layer. For the mono- and bi-layer phosphorene samples with an extra passivation layer of 5 nm ALD Al_2_O_3_ on top of the P_*x*_O_*y*_ layer, we did not observe any decay of the PL decay over 6 days (Fig. 3a) and we could still observe strong PL from those samples even after 2 months (Supplementary Fig. 7). Compared with the samples capped by P_*x*_O_*y*_ alone, those capped with dual layers of P_*x*_O_*y*_ and Al_2_O_3_ have much longer lifetime and still show very clean surfaces even after 2 months in air (Supplementary Fig. 7). Of course, we could improve our current passivation technique (P_*x*_O_*y*_ plus 5 nm of ALD Al_2_O_3_) to obtain even longer sample lifetime. For instance, we could use double capping layers of thick Al_2_O_3_ and hydrophobic fluoropolymer on top of the P_*x*_O_*y*_, which was demonstrated to be a very efficient way to passivate thick phosphorene flakes^24^. Also, instead of using a SiO_2_/Si substrate, we can transfer the initial thick phosphorene flake onto a hydrophobic substrate, such as hexagonal boron nitride^26^ or a fluoropolymer^24^, which should improve the passivation quality from the bottom side. These strategies will be pursued in future studies, but were not the focus of this work.

### Defect engineering in monolayer phosphorene

**Figure 4:**
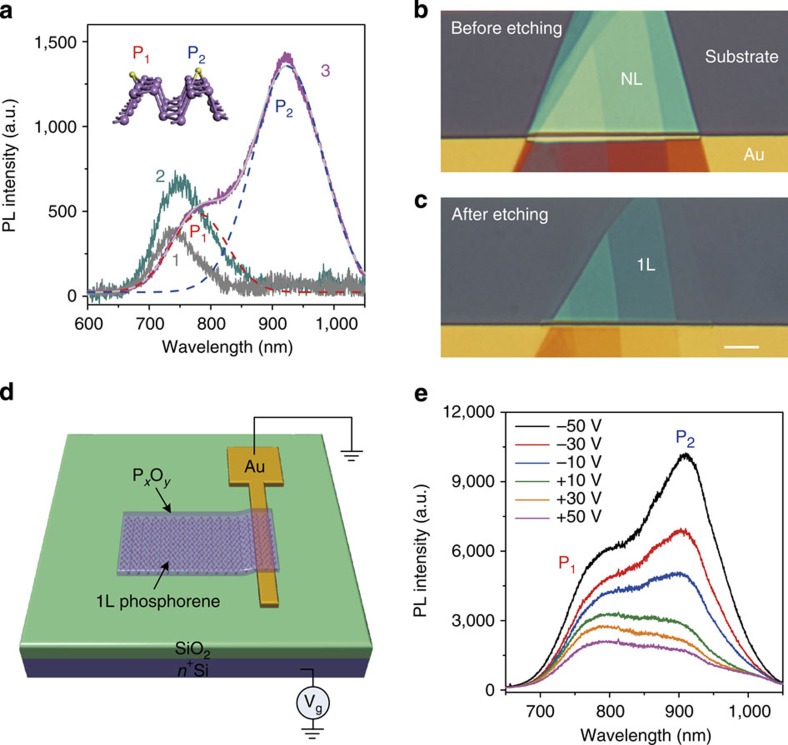
Oxygen defect engineering in stabilized monolayer phosphorene. (**a**) Measured PL spectra of monolayer phosphorene samples produced by different methods: sample 1—exfoliated monolayer phosphorene; sample 2—monolayer phosphorene with P_*x*_O_*y*_ capping layer fabricated by oxygen plasma etching; sample 3—monolayer phosphorene with P_*x*_O_*y*_ capping layer fabricated by oxygen plasma etching, but with ∼2 s' over etching by oxygen plasma compared with sample 2. The spectrum of sample 3 is fit to two Gaussian peaks labeled by P_1_ and P_2_ (dashed red and blue lines show the peaks at 780 and 915 nm, respectively; solid grey line is the cumulative result for the fitting). Inset is schematic plot of two different oxygen defects in monolayer phosphorene (P_1_ —horizontal bridge and P_2_ —diagonal bridge), which were discussed in ref. 27. (**b**,**c**) Optical microscope images showing the fabrication of a monolayer phosphorene metal—oxide—semiconductor (MOS) device, using O_2_ plasma etching. An exfoliated phosphorene flake was dry transferred onto a SiO_2_/Si substrate (275 nm thermal oxide on *n*^+^-doped silicon), in contact with a gold electrode that was pre-patterned on the substrate. Half of the phosphorene flake was on the gold electrode and the other half on the SiO_2_ substrate (**b**). O_2_ plasma etching was then used to thin down the flake to a monolayer (**c**). The over etching process was used to trigger the defect peaks in the so-produced monolayer phosphorene. Scale bar, 10 μm. (**d**) Schematic plot of the monolayer phosphorene MOS structure and the electrical measurement set up. (**e**) Measured PL spectra from the monolayer phosphorene in (**c**), under different back gate voltages from −50 to +50 V.

Defects in semiconductors, such as vacancies and interstitials, can strongly influence transport and optical properties of the host material and such interactions become stronger in low dimensional material due to the tighter localization of the electron wave function^38^. Understanding the functionalities of defects, and the ability to control and manipulate them with atomic precision, can improve the performance of 2D materials and lead to new applications^39^. As predicted by previous simulation work^40^, the defects in monolayer phosphorene can significantly modulate its optical and electrical properties, enabling important and new applications. However, it has been a long-standing challenge to precisely engineer the defects in monolayer phosphorene, due to its fast degradation in ambient condition discussed earlier. Here we found that our O_2_ plasma etching technique offers the opportunity to precisely engineer defects in a stabilized phosphorene monolayer for the first time. A high-quality phosphorene monolayer fabricated by O_2_ plasma etching shows one PL emission peak at 750 nm, which is the same with that from a fresh exfoliated phosphorene monolayer (Fig. 4a). This peak (750 nm) is denoted as the trion (charged exciton) peak of a pristine phosphorene monolayer, in accordance with our previous research^19^. The phosphorene monolayer fabricated by O_2_ plasma etching has a significantly higher PL intensity than the exfoliated sample in the same measurement conditions, which could be due to its high quality and its well-protected surface. Both monolayer phosphorene samples produced by exfoliation and by O_2_ plasma etching (sample 1 and sample 2 in Fig. 4a) show a slightly asymmetric PL spectra profile with a low-energy tail, which is consistent with the charge-recoil effect in trion emissions^41^. The recombination of a trion will emit a photon and leave a free charge behind, which leads to the low-energy tail in the PL spectrum^41^. Please note that sample 1 was put into a Linkam THMS 600 chamber with a slow flow of N_2_ gas for sample protection and the temperature was set as −10 °C also for sample protection, during the PL measurements, similar to that in our previous report^19^; while the PL spectra of sample 2 was measured in air at room temperature. The PL peak from sample 2 has slightly larger full width at half maximum than that from sample 1. This could be because that the full width at half maximum normally becomes larger as temperature increases due to the thermal broadening effect^41^.

We then introduced oxygen defects into the phosphorene monolayer, through controllable over etching with O_2_ plasma. Interestingly, the over etched phosphorene monolayer shows two strong PL peaks at 780 and 915 nm, respectively: the 750 nm peak being significantly diminished. The peak at 915 nm has the highest PL intensity. To understand the origin of these two new peaks, we ran power-dependent PL measurements. The integrated PL of these two peaks grow sub-linearly with the excitation power (*Δ*=0.54 for 780 nm peak and *Δ*=0.58 for 915 nm peak, Supplementary Fig. 8), respectively, indicating that they are from localized excitons^42^,^43^.

These two peaks can be attributed to the localization of excitons induced by oxygen defects, which has been predicted by numerical simulations^40^. On the basis of those simulations^40^, for a phosphorene monolayer, there are two bridge-type surface oxygen defects (diagonal bridge and horizontal bridge) with positive binding energies, and these may be formed if the oxygen source is more reactive than the O_2_ ground state (for example, under light pumping or O_2_ plasma). For a diagonal bridge defect, the oxygen atom connects phosphorus atoms on different edges of the zigzag ridge; for a horizontal bridge defect, the oxygen atom connects the phosphorus atoms from the same edge (Fig. 4a inset). Both types of surface bridge defects create levels in the bandgap. Such gap states are expected to give rise to recombination lines in luminescence experiments. On the basis of the simulated energy diagrams for these two types of bridge defects^40^, we attribute the emission peaks of 780 and 915 nm to the horizontal bridge oxygen defects and the diagonal bridge oxygen defects, respectively. Of course, those two PL peaks are broad and there might contain the contributions from other types of oxygen defects, such as dangling oxygen atoms, which has been discussed in previous simulation work^40^. The measured temperature-dependent peak positions of the two PL peaks P1 and P2 do not follow the standard semiconductor temperature-dependence bandgap equation (Supplementary Fig. 9 and Supplementary Note 5), which could be due to potential fluctuation and band tail states that lead to exciton localization^44^,^45^,^46^.

Localized exciton emissions for monolayer transition-metal dichalcogenide semiconductors can only be seen at cryogenic temperatures^39^. In contrast, the emission from oxygen defects in a phosphorene monolayer can be clearly observed at room temperature, which could be due to their large trapping energy^40^. Here we further demonstrate that the PL intensity and spectra from these defects can be tuned by an electrical gate, which could lead to new applications as electrically tunable and broadband lighting devices in the near infrared range at room temperature. In these experiments, a mechanically exfoliated phosphorene flake was dry transferred^47^ onto a SiO_2_/Si substrate (275 nm thermal oxide on *n*^+^-doped silicon), in contact with a gold electrode that was pre-patterned on the substrate. Half of the phosphorene flake was on the gold electrode and the other half on the SiO_2_ substrate (Fig. 4b). O_2_ plasma etching was then used to thin down the flake to a monolayer (Fig. 4c), and PSI and PL spectral measurements were used to confirm the layer number (Supplementary Fig. 10). We then intentionally over etched the sample by ∼2 s to induce the oxygen defects and the defect-induced PL peaks emerged. In the measurement, the gold electrode was grounded, and the *n*^+^-doped Si substrate was used as a back gate providing a uniform electrostatic doping for the phosphorene monolayer in this metal–oxide–semiconductor (MOS) structure (Fig. 4d). The measured PL spectrum was very sensitive to the electrostatic doping and can be significantly modulated by the gate voltage. Under each gate bias, the PL emission showed a broad spectrum ranging from ∼750 to 950 nm which could be fitted by the two oxygen defect-induced peaks at 780 and 915 nm. As we gradually swept the back gate voltage from 50 to −50 V, the intensities of those two defect peaks at 780 and 915 nm were enhanced by a factor of 2.5 and 5.0, respectively, whilst the positions of the peaks were not sensitive to the gate voltage (Supplementary Fig. 11). The different enhancement factors for the two peaks allows us to modulate the shape of the spectrum in the near infrared. This difference in the enhancement factors could be due to the initial Fermi level in the doped phosphorene monolayer being close to the lower energy defect states^40^. The PL intensity is enhanced when we inject positive charge into the sample, suggesting that the phosphorene monolayer with oxygen defects has an initial *n*-type doping. As we know, pristine phosphorene flakes have *p*-type doping owing to the initial pristine defect dopants in a bulk black phosphorus crystal^1^,^11^. This means that the oxygen defect engineering process converted the phosphorene monolayer from initial *p*- to *n*-type, since oxygen dopants are *n*-type for phosphorene crystals. This oxygen defect engineering technique can lead to new electronic and optoelectronic devices, since it not only significantly influences the optical properties of a phosphorene monolayer, but can also engineer its doping.

## Discussion

We were able to control the defect concentration in the phosphorene samples by controlling the over-etching time, and this allowed us to tune both the PL and Raman spectra for a phosphorene monolayer (Supplementary Fig. 12 and Supplementary Note 6). A small amount of oxidation triggered the defect PL emissions; and with further oxidation, the intensity of the defect PL peak dropped, because the monolayer phosphorene was eventually etched away (Supplementary Fig. 12a). The integrated intensity ratio A_g_^1^/A_g_^2^ dropped significantly as we increased the over etching time (Supplementary Figs 12b and c), which further confirms the relationship between the intensity ratio A_g_^1^/A_g_^2^ and the oxidation-induced defect concentration in a phosphorene monolayer^29^.

Please note that the peak PL intensity of 1L phosphorene is normally lower than that of 2L phosphorene, for both our exfoliated samples and the samples produced by O_2_ plasma etching. This might be due to that the PL spectra of 1L phosphorene was recorded by charge-coupled device (CCD) detector, while the PL spectra of 2–4L phosphorene were recorded by InGaAs detector. The optics and efficiency for those two detectors are different, which makes it hard to compare the PL intensity from 1L phosphorene with others (Supplementary Fig. 13).

In conclusion, we demonstrate a new method to controllably fabricate air-stable and high-quality phosphorene samples with a designated number of layers, from a few down to a monolayer. This technique opens new opportunities for the synthesis of high-quality phosphorene samples, enabling a wide range of fundamental research and device applications. Moreover, the stabilized phosphorene monolayer provides us with a perfect platform to precisely engineer the defects, which results in new photon emissions with high efficiency in the near infrared range at room temperature. Furthermore, we successfully used an electrostatic gate to significantly tune the defect-triggered photon emission for a phosphorene monolayer. This capability to manipulate the defects can lead to new electronic and optoelectronic devices, such as electrically tunable and broadband lighting devices in the near infrared at room temperature.

## Methods

### Phosphorene sample fabrication by oxygen plasma etching

A thick phosphorene flake was mechanically exfoliated to a 275-nm SiO_2_/Si substrate and then the sample was treated under O_2_ plasma etching by Oxford Plasmalab 100 ICP-RIE system. The O_2_ plasma etching used 400 W ICP generator power, 30 W RF bias power, with 30 s.c.c.m. O_2_ flow at 10 mTorr pressure. The O_2_ plasma pre-treatment process took 40 s then the P_*x*_O_*y*_ layer reached a constant thickness of ∼11 nm and phosphorene etching rate also became constant at ∼10 s per layer.

### MOS device fabrication and characterizations

For the MOS structure, we used mechanical exfoliation to dry transfer^47^ a phosphorene flake onto a SiO_2_/Si substrate (275 nm thermal oxide on *n*^+^-doped silicon), with half on a pre-patterned gold electrode half on substrate. The gold electrodes were patterned by conventional photolithography, metal deposition and lift-off processes. All the OPL characterizations were obtained using a phase-shifting interferometer (Vecco NT9100). All Raman and PL spectrum measurements were conducted using a T64000 micro-Raman system equipped with a CCD and InGaAs detectors, along with a 532 nm Nd:YAG laser as the excitation source. PL spectra of 1L phosphorene was recorded by a Si CCD detector, while the PL spectra of 2–4L phosphorene were recorded by an InGaAs detector. During the PL measurements, the exfoliated phosphorene monolayer samples were put into a Linkam THMS 600 chamber with a slow flow of N_2_ gas for sample protection and the temperature was set as −10 °C, similar to that in our previous report^19^. The PL spectra of the 1–4L phosphorene samples produced by O_2_ plasma etching was measured in air at room temperature. Integrated PL mapping image (over the spectral range of 715–1095, nm) was generated using a commercial WiTec alpha300S system equipped with an avalanche photodiode in scanning confocal microscope configuration. The electrical bias was applied using a Keithley 4200 semiconductor analyzer.

### Numerical simulation

The Stanford Stratified Structure Solver (S4) was used to calculate the phase delay. The method numerically solves Maxwell's equations in multiple layers of structured materials by expanding the field in the Fourier space.

## Additional information

**How to cite this article:** Pei, J. *et al*. Producing air-stable monolayers of phosphorene and their defect engineering. *Nat. Commun.* 7:10450 doi: 10.1038/ncomms10450 (2016).

## Supplementary Material

Supplementary Information Supplementary Figures 1-13, Supplementary Notes 1-6 and Supplementary References
