# Supplementary material for: Producing air-stable monolayers of phosphorene and their defect engineering
Source: Nat Commun. 2016 Jan 22;7:10450. doi: 10.1038/ncomms10450 (PMC4735856; doi:10.1038/ncomms10450)
Supplement: Supplementary Information — Supplementary Figures 1-13, Supplementary Notes 1-6 and Supplementary References [file ncomms10450-s1.pdf]

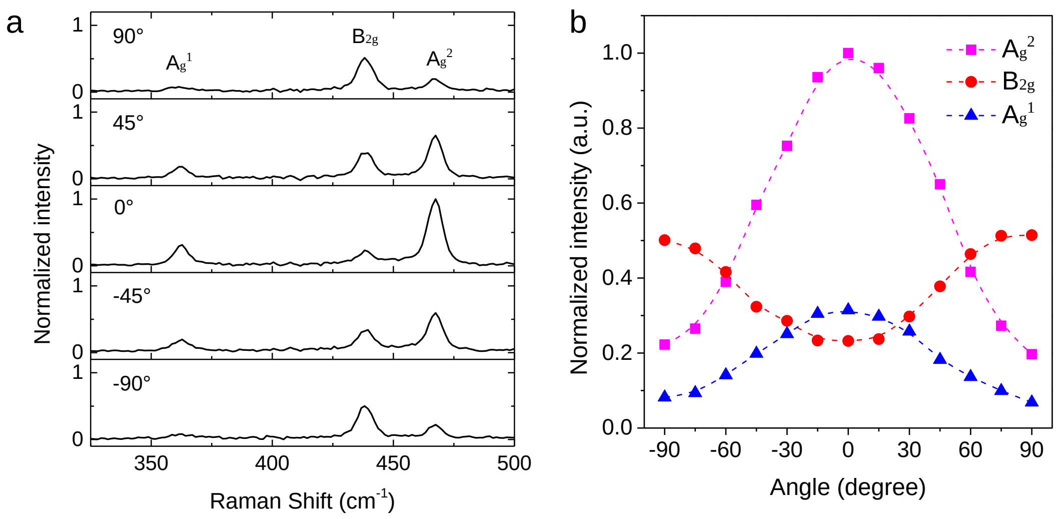

Supplementary Figure 1 | a, Measure Raman spectra of a monolayer phosphorene produced by O_2 plasma etching, under different polarization angles. **b,** Polarization dependence of A_g^1, B_{2g}, and A_g^2 modes.

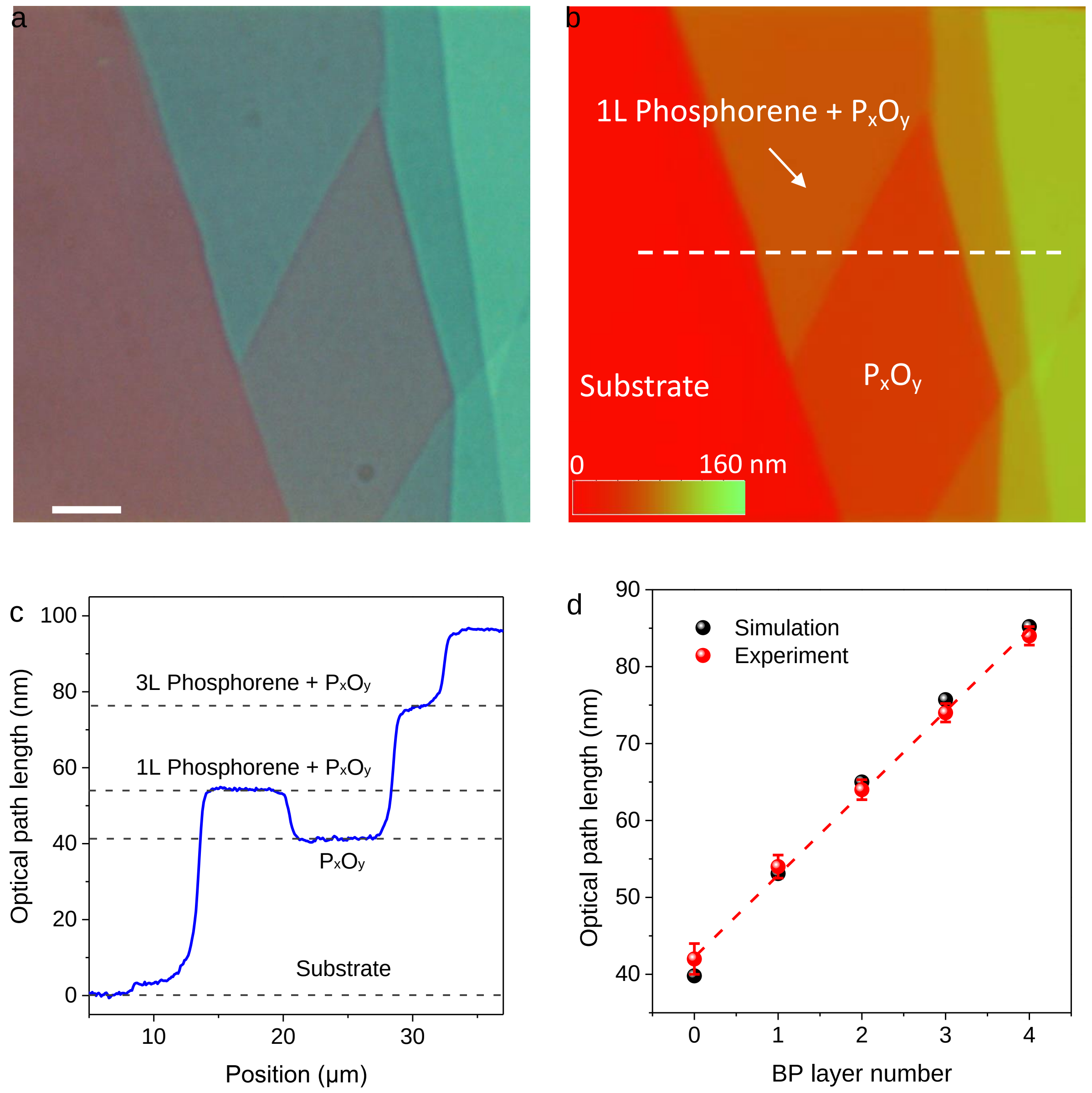

Supplementary Figure 2 | Identification by phase-shifting interferometry (PSI) of the layer number of the phosphorene samples produced by O_2 plasma etching. a-b, Optical microscope (a) and PSI (b) images of a phosphorene flake etched by O_2 plasma, which generates a monolayer phosphorene sample capped with P_xO_y and another pure P_xO_y flake. Scale bar, 5 μm. **c,** PSI measured optical path length (OPL) values versus position along the dashed line in (b). **d,** OPL values from simulation and experiment PSI measurements for 0L, 1L, 2L, 3L and 4L phosphorene/P_xO_y stacks. 0L phosphrene/P_xO_y stack means the phosphorene flake is just fully etched and only P_xO_y exists. For each layer number, at least three different samples were characterized for the statistical measurements. The red dashed line is the linear trend for statistical data measured with the PSI system.

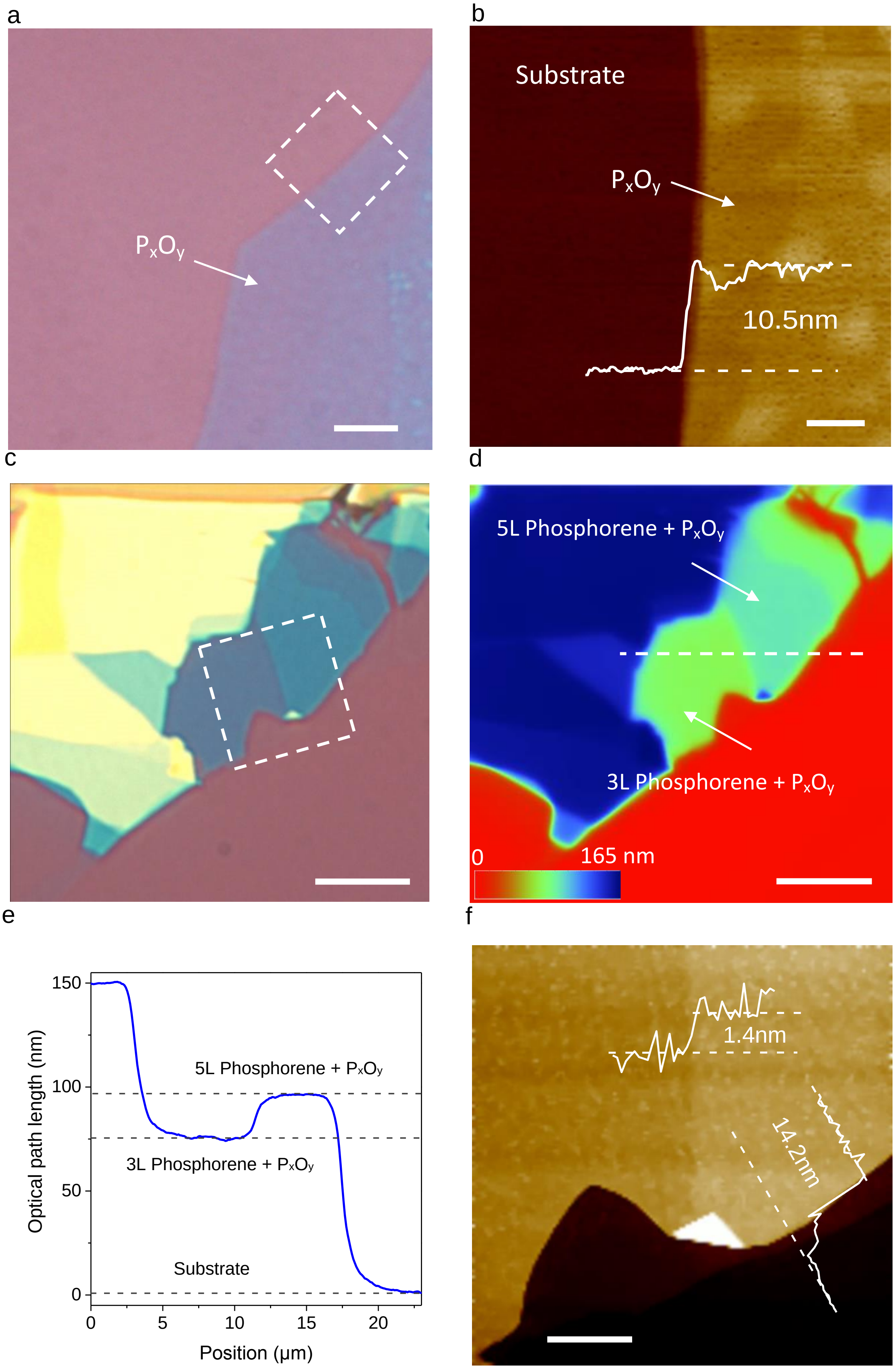
a
P_xO_y
b
Substrate
P_xO_y
10.5nm
c
d
5L Phosphorene + P_xO_y
3L Phosphorene + P_xO_y
0
165 nm
e
150
100
50
0
0
5
10
15
20
Optical path length (nm)
Position (μm)
5L Phosphorene + P_xO_y
3L Phosphorene + P_xO_y
Substrate
f
1.4nm
14.2nm

Supplementary Figure 3 | Determining the thickness of phosphorene and P_xO_y by PSI and atomic force microscopy (AFM). a, Optical microscope image of a pure P_xO_y flake created by O_2 plasma etching, when the phosphorene layer was just fully etched. Scale bar, 5 µm. **b,** AFM image of the pure P_xO_y marked by a dashed rectangle in (a). Scale bar, 1 µm. **c,** Optical microscope image of an O_2 plasma etched flake with 3L and 5L phosphorene that are capped with P_xO_y. Scale bar, 8 µm. **d,** PSI image of the P_xO_y capped 3L and 5L phosphorene. Scale bar, 8 µm. **e,** PSI measured optical path length (OPL) values versus position along the dashed line in (d). **f,** AFM image of the area marked by a dashed rectangle in (c). Scale bar, 2 µm.

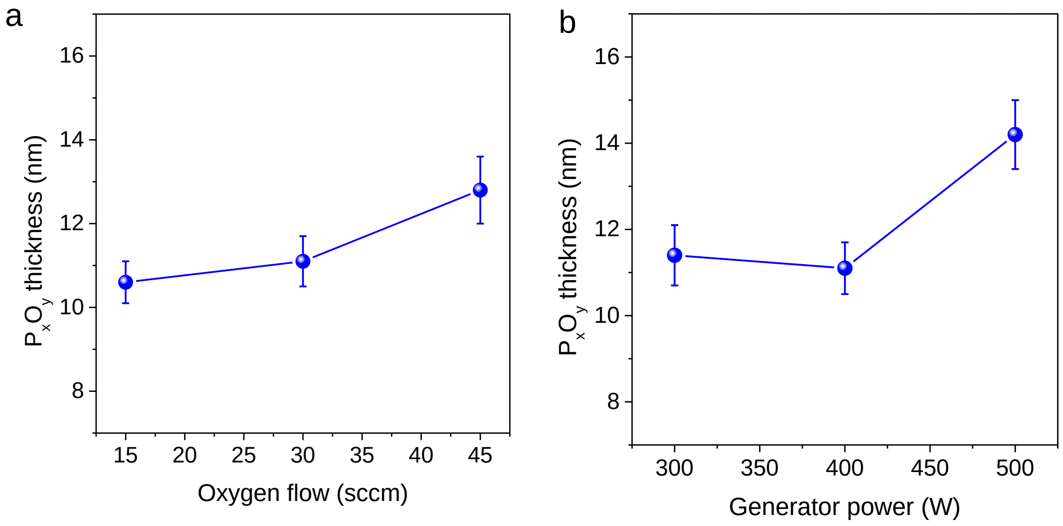

Supplementary Figure 4 | a, Measured P_xO_y thickness as a function of oxygen flow rate, when the power was fixed at 400 W. **b,** Measured P_xO_y thickness as a function of generator power, when the oxygen flow rate was fixed at 30 sccm. The error bar represents the statistical error from at least two samples under the same plasma condition.

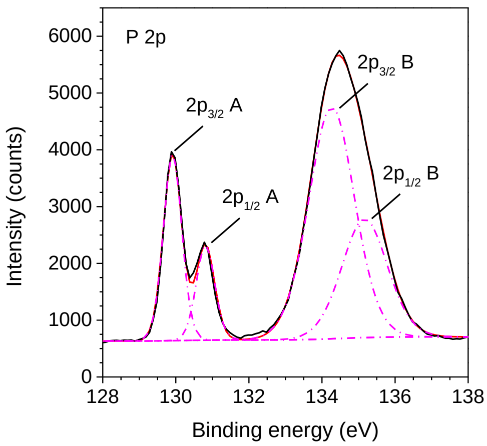

Supplementary Figure 5 | X-ray photoelectron spectroscopy spectra in O_2 plasma etched black phosphorus sample. It shows the spectra of the P *2p* core level. $2p_{3/2}$ A and $2p_{1/2}$ A represent *P-P* bonds of black phosphorus; $2p_{3/2}$ B and $2p_{1/2}$ B is for saturated-oxidized P_2O_5.

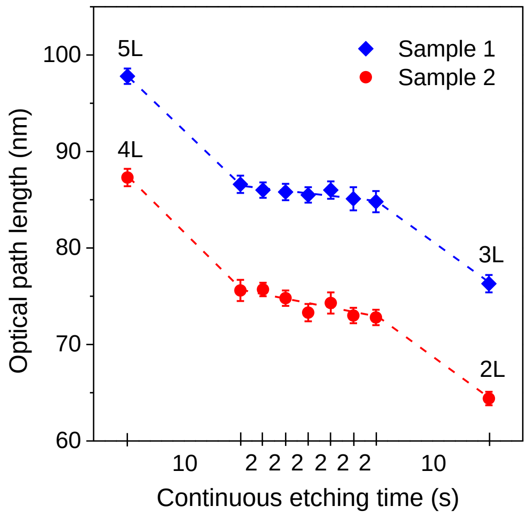

Supplementary Figure 6 | “Step-wise” etching of phosphorene flakes by O_2 plasma. The error bar represents the measured thickness variation from different locations of the same stack.

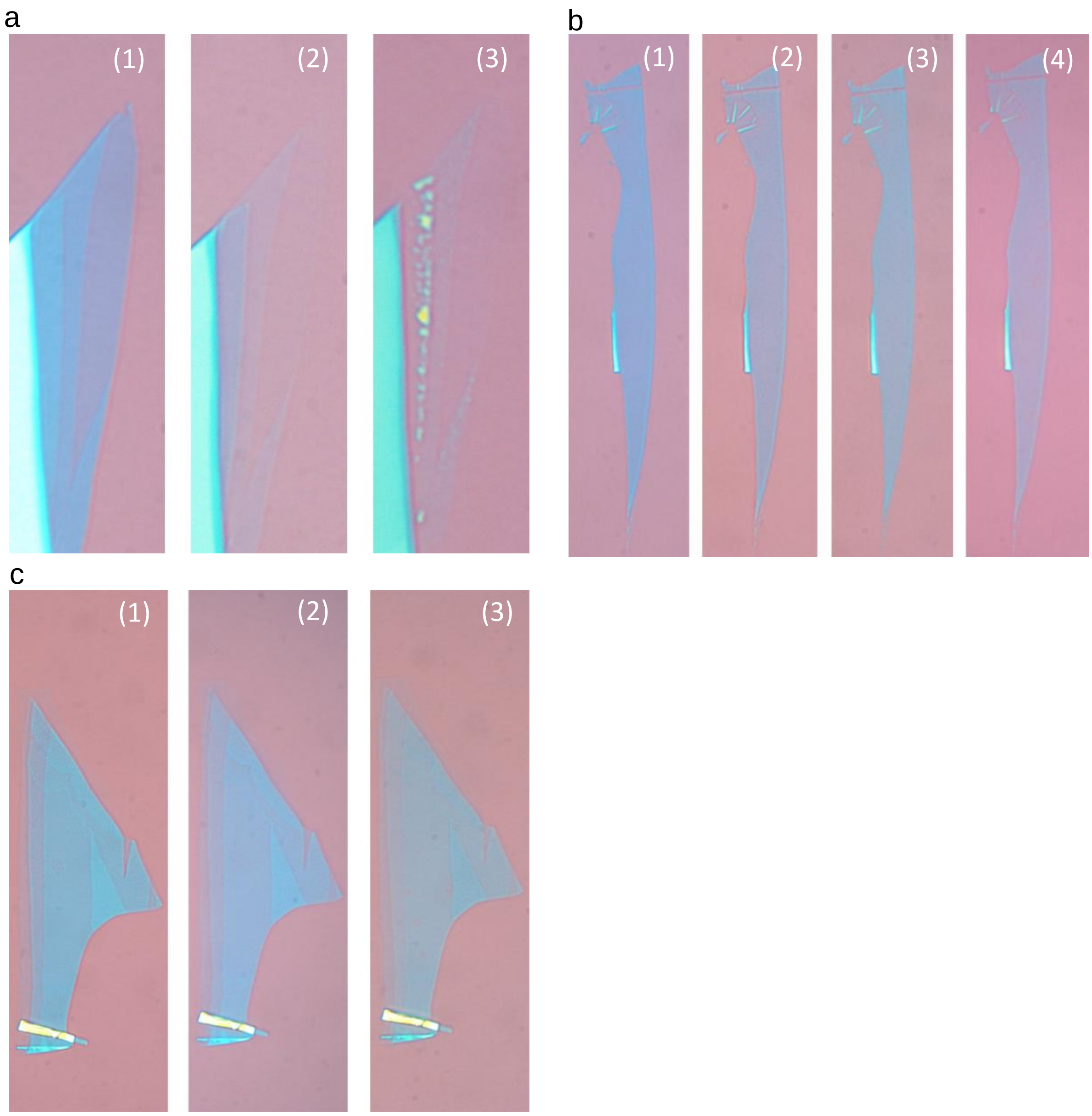

Supplementary Figure 7 | Comparison of the ALD Al_2O_3passivation on phosphorene samples created by exfoliation and O_2 plasma etching. a, Optical microscope images of an exfoliated 2L phosphorene sample, as just exfoliated before ALD (1), after 5 nm of ALD Al_2O_3 passivation (2) and after two weeks in ambient condition (3). **b,** Optical microscope images of a 2L phosphorene sample with capping P_xO_y layer produced by O_2 etching, as just fabricated before ALD (1), after 5 nm of ALD Al_2O_3 passivation (2), after one month in ambient condition (3) and after two months in ambient condition (4). **c,** Optical microscope images of the phosphorene sample shown in main figure 3d-e, after 5 nm of ALD Al_2O_3 passivation (1), after one month in ambient condition (2) and after two months in ambient condition (3).

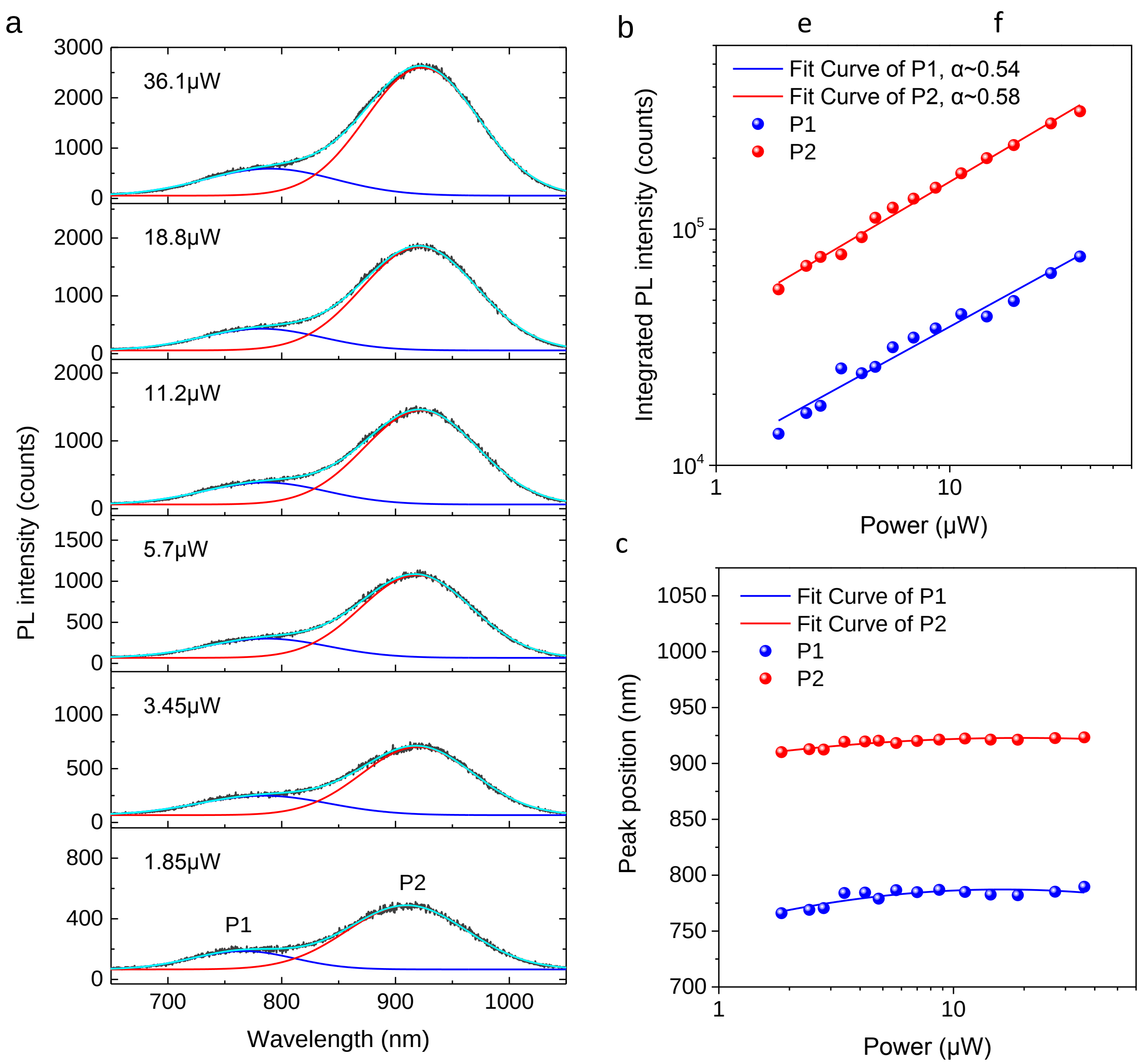

Supplementary Figure 8 | Power dependence analysis of defect-induced photon emissions in 1L phosphorene sample produced by O_2 plasma etching. **a**, Measured PL spectra (gray lines) with various excitation power. Each PL spectrum is fit to two Gaussian peaks, labeled as P1 (blue line) and P2 (red line). Cyan lines are the cumulative fitting results. **b**, Log-log plots of integrated PL intensity of peaks P1 and P2 as a function of laser power. From the fitting curves, integrated PL of P1 and P2 both grow sub-linearly with the excitation power, with a slope of α = 0.54 and 0.58 for P1 and P2, respectively. **c**, PL peak position of P1 and P2 as a function of excitation power.

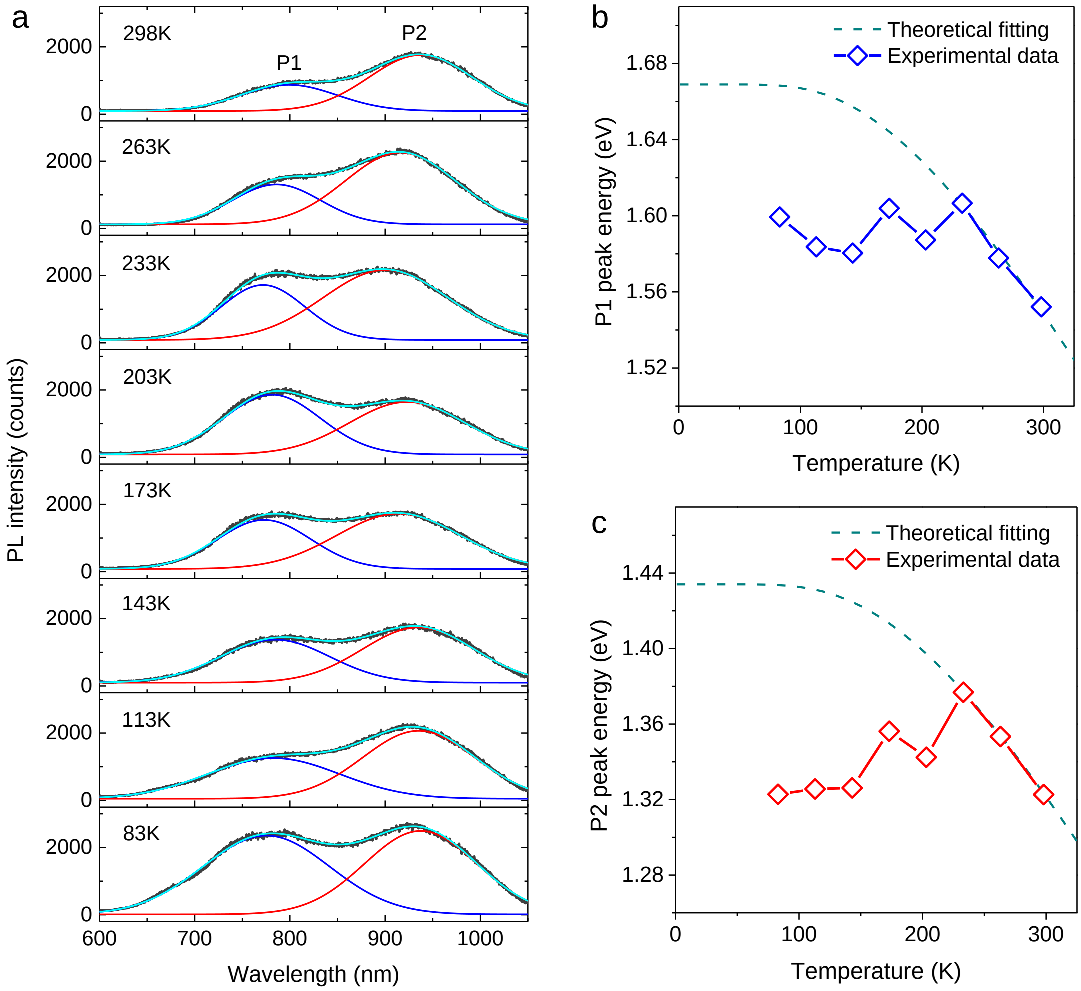

Supplementary Figure 9 | a, Measured PL spectra (grey lines) for a monolayer phosphorene sample produced by O_2 plasma etching with defect engineering, at temperature ranging from 83 to 298 K. Each PL spectrum is fit to two Gaussian peaks, labeled as P1 (blue line) and P2 (red line). P1 and P2 have been attributed to localized excitons. **b-c,** Temperature dependence of PL peak energies of P1 (b) and P2 (c). These two emission peaks show an "S-shaped" shift behavior with increasing temperature. The solid lines in (b) and (c) are the experimental data. The dotted lines are the fit of data using a standard semiconductor bandgap dependence of $E_g(T) = E_g(0) - S\hbar\omega[\coth(\frac{\hbar\omega}{2kT}) - 1]$, where $E_g(0)$ is the ground-state transition energy at 0 K, S is a dimensionless coupling constant and $\hbar\omega$ is an average phonon energy. From the fits, we extract the parameters for P1 (P2) to be E_g = 1.669 (1.434) eV, S = 7.482 (7.816) and $\hbar\omega$ = 51.83 (56.27) meV.

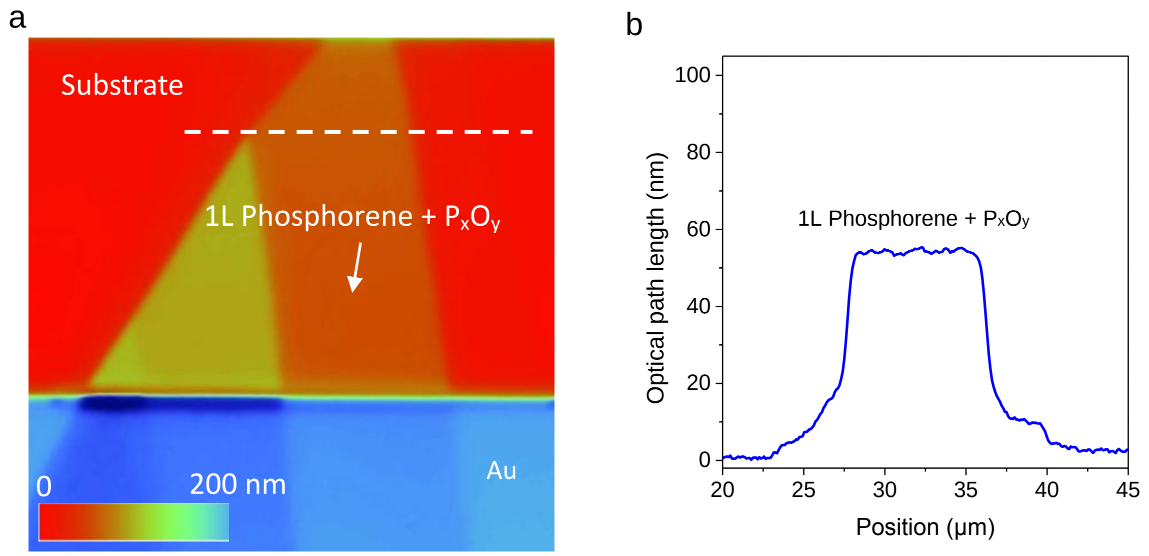

Supplementary Figure 10 | a, PSI image of monolayer phosphorene with capping P_xO_y layer created by O_2 plasma etching, as shown in Figure 4c. **b,** Optical path length of the 1L phosphorene/P_xO_y stack marked by the dashed line in (a).

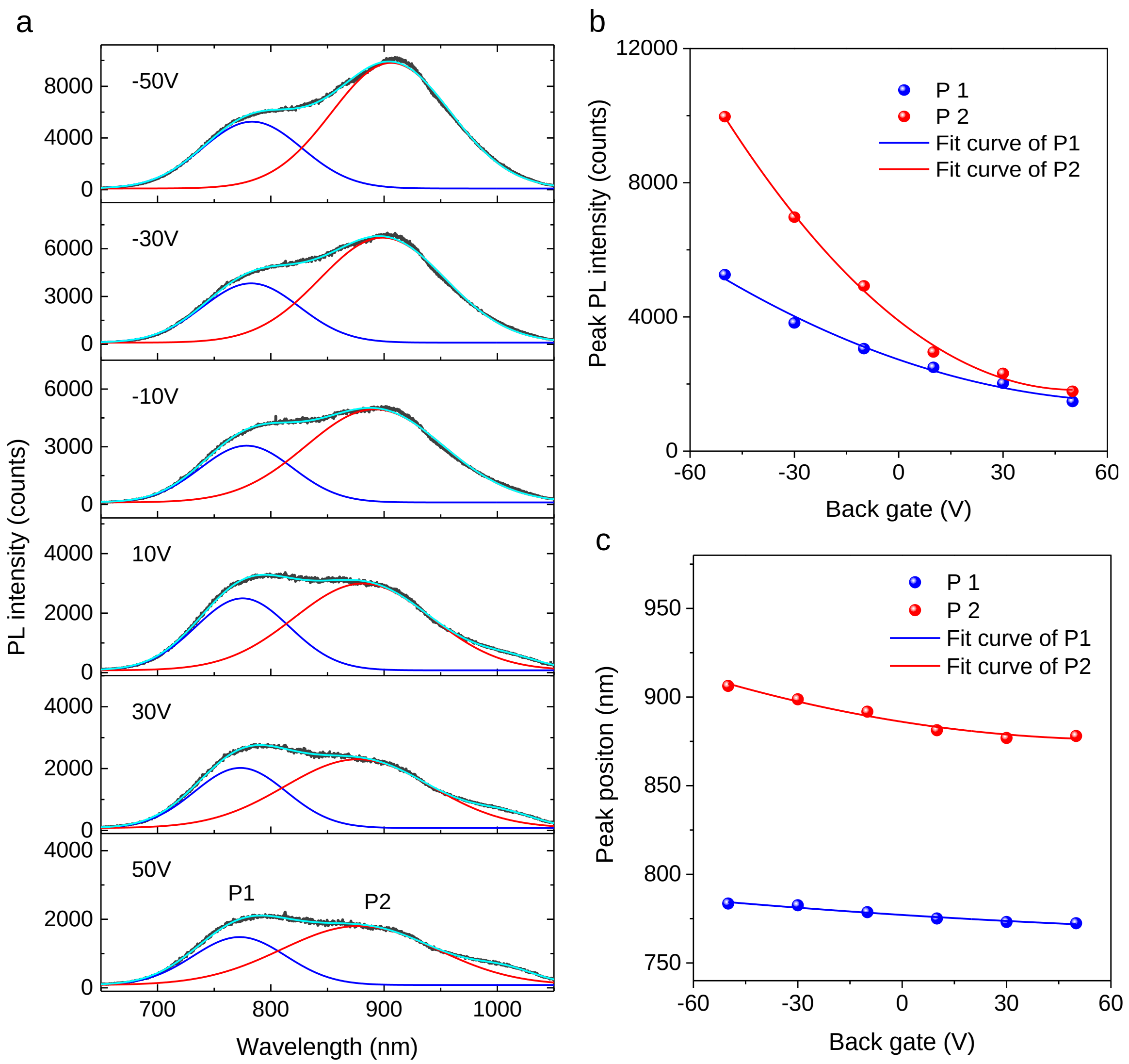

Supplementary Figure 11 | Gate dependence of the defect-triggered photon emissions in the 1L phosphorene MOS device (Figure 4d-e), produced by O_2 plasma etching. a, Measured PL spectra (gray lines) under various back gate voltages. PL spectra are fit to Gaussians (blue lines are labeled as peak P1, red lines are labeled as peak P2, and cyan lines are the cumulative fitting results). **b**, Integrated PL intensity of P1 and P2 as a function of back gate biases. **c**, PL peak position of P1 and P2 as a function of back gate biases. Solid lines are the polynomial fit curves.

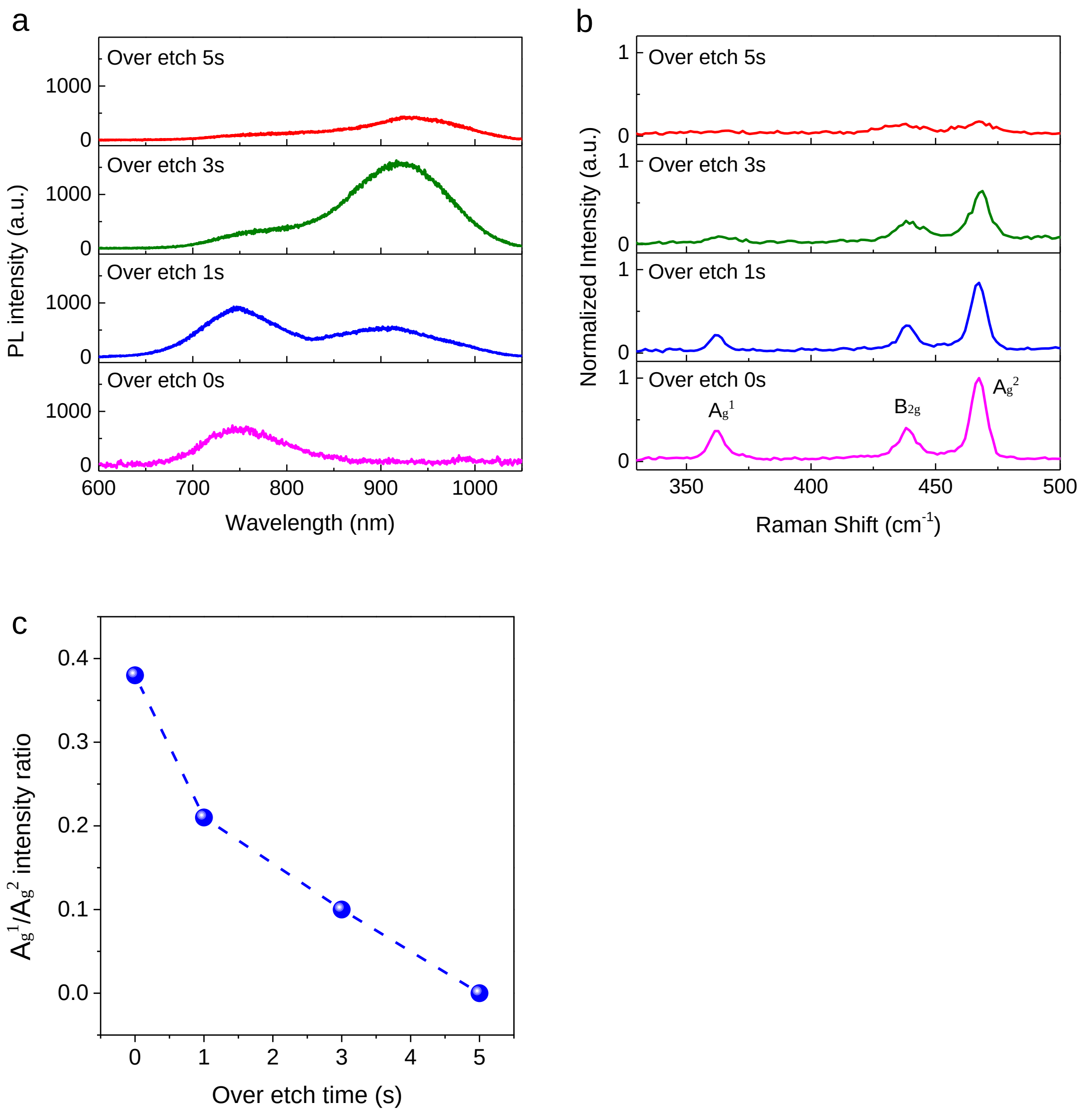

Supplementary Figure 12 | a-b, Measured PL (a) and Raman spectra (b) from monolayer phosphorene samples produced by O_2 plasma etching, at different over etching time of 0, 1, 3 and 5 seconds, respectively. The over etching was carried out through a continuous etching of a (2L phosphorene + P_xO_y) stack at a total time of 10 seconds plus the over etching time. **c,** The integrated peak ratio A_g^1/A_g^2 from Raman spectra (b) of the monolayer phosphorene, as a function of over etching time.

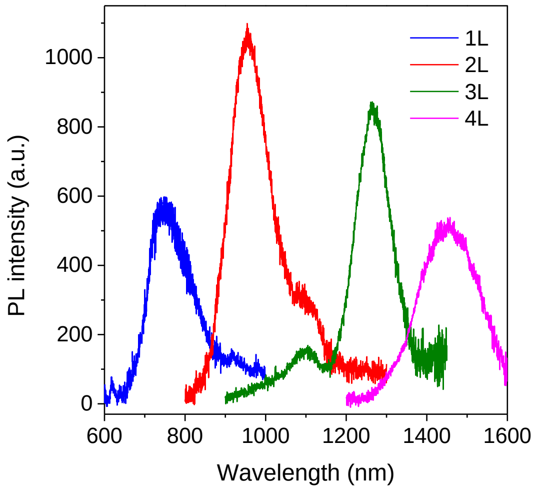

Supplementary Figure 13 | Measured photoluminescence (PL) spectra from the thin phosphorene samples (quad- to mono-layer, 4-1L), fabricated by O_2 plasma etching.

Supplementary Note 1

The strongly angle dependent responses of the A_g^1, B_{2g} and A_g^2 modes confirms the anisotropic puckered crystalline structure of high quality monolayer phosphorene samples produced by our etching method[1]. Compared with single-crystalline bulk black phosphorus, amorphous black phosphorus[2] shows a largely shifted A_g^2 Raman mode at ~480 cm^{-1}. Our phosphorene samples produced by O_2 plasma etching do not have this largely shifted Raman mode at ~480 cm^{-1}, which suggests that there is no amorphous black phosphorus in our samples (Supplementary Fig. 1).

Supplementary Note 2

For the pure P_xO_y, its physical thickness was measured to be ~10.5 nm by AFM (Supplementary Fig. 3a and 3b), which agrees well with the extracted value of ~11 nm from our PSI measurements and fitting. Supplementary Fig. 3c-f show the characterization images for another flake that was produced by O_2 etching. Based on our PSI measurements, this flake has a stack of (3L phosphorene + P_xO_y) and another stack of (5L phosphorene + P_xO_y) (Supplementary Fig. 3d & 3e). Then we used AFM to determine the physical thicknesses of those two stacks. The (5L phosphorene + P_xO_y) stack shows an AFM thickness of 14.2 nm. Also, there is a thickness difference of 1.4 nm between the stacks of (5L phosphorene + P_xO_y) and (3L phosphorene + P_xO_y). This means the phosphorene has a physical thickness of ~0.7 nm per layer, which is consistent with previous reports[3, 4]. Then the physical thickness of P_xO_y in this piece is determined to be ~10.7 nm, which matches well with the AFM thickness of P_xO_y in Supplementary Fig. 3b.

Supplementary Note 3

Based on our experiments, under a fixed generator power of 400 W, the P_xO_y thickness slightly increased from 10.5 to 12.8 nm, when we increased oxygen flow rate from 15 to 45 sccm (Supplementary Fig. 4). This could be because that the phosphorene oxidation rate is enhanced more than the enhancement of the sputtering rate as the oxygen flow rate is

increased which leads to a new equilibrium state that has thicker P_xO_y. When the oxygen flow rate was fixed at 30 sccm, the measured P_xO_y thickness did not change monotonically with the increase of the generator power. Of course, more experimental work is needed to completely understand the physics and parameter dependence which will be the subject of further research.

Supplementary Note 4

We found that the O_2 plasma results in step-wise etching. We tested two samples. Sample #1 was an O_2 plasma etched 5L phosphorene layer capped with P_xO_y. Then sample #1 was further thinned to (4L BP + P_xO_y), by a 10 second long cycle of continuous etching. Next, the (4L BP + P_xO_y) stack was etched for 2 seconds by the O_2 plasma, which did not lead to significant thickness change. Then five more times of 2 seconds' O_2 etching treatment was appled, with the thickness probed after each of the 2 second treatment. We found that the thickness of the (4L BP + P_xO_y) stack remained almost constant even after the six 2 second treatments. Finally, a 10 seconds continuous O_2 etching was applied, and this led to the thinning of sample down to (3L BP + P_xO_y). Sample #2 also showed similar "step-wise" etching (Supplementary Fig. 6). We believe that this type of "step-wise" etching is related to the diffusion kinetics of oxygen plasma in P_xO_y during the O_2 plasma process, which is similar to that for the oxidation of crystalline Si in an O_2 plasma[5, 6]. In O_2 plasma etching, the oxygen plasma diffuses through the growing oxide, whilst the plasma is present. In our experiments, the oxygen plasma needs several seconds (> 5) to diffuse from the P_xO_y-plasma interface to the BP-P_xO_y interface. If the plasma is applied for only 2 seconds, the oxygen diffuses only a small distance into the P_xO_y layer and does not cause any oxidation of the phosphorene layer. Once the plasma is off, oxygen can diffuses out of the P_xO_y layer, because of the high vacuum in the chamber. This explains why etching using repeated 2-second cycles does not accumulate.

Supplementary Note 5

The solid lines in Supplementary Fig. 9b and 9c show an "S-shaped" emission shift behaviour with increasing temperature. This type of "S-shaped" temperature dependence is

commonly attributed to potential fluctuation and band tail states that lead to exciton localization[7-9]. In the high temperature region, the PL peak energies blue shift as temperature decreases, which follows the temperature dependence described by the standard semiconductor bandgap dependence equation[10] of $E_g(T) = E_g(0)\text{-}S\hbar\omega[\coth(\frac{\hbar\omega}{2kT})\text{-}1]$, where $E_g(0)$ is the ground-state transition energy at 0 K, S is a dimensionless coupling constant and $\hbar\omega$ is an average phonon energy. The PL peak energies decrease as the temperature is further decreased. This anomalous red shift is attributed to the transfer and filling processes of the band tail states or localized states[7-9]. Finally, at low temperatures, the PL peak energies can blue shift again (Supplementary Fig. 9b) with decreasing temperature, which is due to the temperature induced bandgap widening. Similar phenomenon was also observed in InGaN/GaN quantum wells[8, 9]. The temperature dependence data further confirm our previous assignments that those two PL peaks P1 and P2 are from localized excitons.

Supplementary Note 6

Although there is no defect peak in the Raman spectra of phosphorene, the integrated intensity ratio A_g^1/A_g^2 reflects the oxidation-induced defect density in phosphorene samples[11]. For instance, the ratio A_g^1/A_g^2 of a 3L BP flake decreases gradually from ~0.3 of its pristine state down to ~0.15 after 120 min exposure in ambient condition[11]. More importantly, pristine monolayer phosphorene (no oxidation) has a ratio A_g^1/A_g^2 of ~0.4, while the partially oxidized one has a ratio value of smaller than 0.2. Our monolayer phosphorene samples produced by O_2 plasma have integrated intensity ratio A_g^1/A_g^2 values in the range of 0.37-0.42, which is comparable to the value of the pristine monolayer phosphorene prepared in glovebox[11]. It indicates the high quality of our monolayer phosphorene samples produced by O_2 plasma etching. Also, this ratio value drops significantly when we increase the over etching time (Supplementary Fig. 12). This is consistent with previous report[11]. From the measured PL spectra, we could clearly see that small amount of oxidation can trigger the defect PL emission peaks. With further oxidation, the intensity of the defect PL peak will drop, because the monolayer phosphorene starts to be etched away.

Supplementary References

1. Ribeiro HB, *et al.* Unusual Angular Dependence of the Raman Response in Black Phosphorus. *ACS Nano* **9**, 4270-4276 (2015).

2. Yang Z, *et al.* Field-Effect Transistors Based on Amorphous Black Phosphorus Ultrathin Films by Pulsed Laser Deposition. *Adv Mater* **27**, 3748-3754 (2015).

3. Liu H, *et al.* Phosphorene: An Unexplored 2D Semiconductor with a High Hole Mobility. *ACS Nano* **8**, 4033-4041 (2014).

4. Zhang S, *et al.* Extraordinary Photoluminescence and Strong Temperature/Angle-Dependent Raman Responses in Few-Layer Phosphorene. *ACS Nano* **8**, 9590-9596 (2014).

5. Martinet C, Devine RAB, Brunel M. Oxidation of crystalline Si in an O2 plasma: Growth kinetics and oxide characterization. *J Appl Phys* **81**, 6996-7005 (1997).

6. Martinet C, Devine RAB. Low‐temperature oxidation of Si in a microwave electron cyclotron resonance excited O_2 plasma. *Appl Phys Lett* **67**, 3500-3502 (1995).

7. Fan S, *et al.* Optical investigation of strong exciton localization in high Al composition $Al_xGa_{1-x}N$ alloys. *Opt Express* **21**, 24497-24503 (2013).

8. Cho Y-H, *et al.* “S-shaped” temperature-dependent emission shift and carrier dynamics in InGaN/GaN multiple quantum wells. *Appl Phys Lett* **73**, 1370-1372 (1998).

9. Hong C-C, Ahn H, Wu C-Y, Gwo S. Strong green photoluminescence from $In_xGa_{1-x}N$/GaN nanorod arrays. *Opt Express* **17**, 17227-17233 (2009).

10. O’Donnell KP, Chen X. Temperature dependence of semiconductor band gaps. *Appl Phys Lett* **58**, 2924-2926 (1991).

11. Favron A, *et al.* Photooxidation and quantum confinement effects in exfoliated black phosphorus. *Nat Mater* **14**, 826-832 (2015).
